# Dissociation between 2-[^18^F]fluoro-2-deoxy-D-glucose positron emission computed tomography, ultrasound and clinical assessments in patients with non-severe rheumatoid arthritis, including remission

**DOI:** 10.1186/s41927-021-00196-1

**Published:** 2021-08-04

**Authors:** Charline Rinkin, Pacôme Fosse, Olivier Malaise, Nathalie Chapelier, Jil Horrion, Laurence Seidel, Adelin Albert, Roland Hustinx, Michel G. Malaise

**Affiliations:** 1 Department of Rheumatology, University Hospital of Liège, Room 155 BC + 3, CHU Sart-Tilman B35, Avenue de l’hôpital 1, B-4000 Liège, Belgium; 2 Department of Nuclear Medicine, University Hospital of Liège, Angers, France; 3 Department of Radiology, University Hospital of Liège, Liège, Belgium; 4 Department of Biostatistics, University Hospital of Liège, Liège, Belgium; 5 Department of Nuclear Medicine, University Hospital of Liège, Liège, Belgium

**Keywords:** Positron emission tomography (PET), Remission, Rheumatoid arthritis (RA), Ultrasonography (US)

## Abstract

**Background:**

Inflammation of patients joints with severe disease activity of rheumatoid arthritis (RA) has already been visualized and quantified by 2-[^18^F]fluoro-2-deoxy-D-glucose positron emission computed tomography ([^18^F] FDG PET/CT), but little is known about the metabolic status and its relationship with clinical and ultrasonography (US) metrology in patients with low/moderate activity or in remission.

**Methods:**

Clinical assessments [based on 28-joint disease activity score (DAS_28_-CRP) and Clinical Disease Activity Index (CDAI)], [^18^F] FDG PET/CT, US and X-ray were performed on 63 RA patients classified into remission or low/moderate or severe disease activity groups. PET/CT was visually and then semi-quantitatively analysed by determining the standardized uptake value (SUV) of positive joints.

**Results:**

Of the 1764 joints, 21.1% were tender only, 13.7% swollen only, 27.6% tender or swollen, 7.3% tender and swollen, 20.5% PET/CT-positive and 8.6% US-positive. PET and US measurements were correlated, albeit with poor concordance. The positive predictive value of PET/CT for clinical evaluation (tender and/or swollen) was low, whereas its negative predictive value was high. Highly significant differences were found with the number of PET/CT-positive joints and with cumulative SUV between “severe” and “non-severe” patients (including those in remission and those with low/moderate activity) and not between those classified as “remission” and “non-remission” or “remission” and “low/moderate activity”. Moreover, the correlation between PET/CT measurements and clinical activity was positive only in the CDAI severe disease group. In patients in remission or with low/moderate activity, only 20–30% of joints were PET/CT-negative. In remission, PET/CT and US were positive in different joints, and PET/CT-positive but US-negative joints mainly exhibited RA (38.1%) or normal (49.2%) and not osteoarthritic (12.7%) X-ray patterns.

**Conclusions:**

[^18^F] FDG PET/CT was effective at distinguishing patients with severely active disease from other patients. In non-severe RA patients, including those in remission, PET/CT results are discordant from US and clinical observations. A longitudinal analysis is needed to explore the clinical relevance of such infra-clinical disease.

**Supplementary Information:**

The online version contains supplementary material available at 10.1186/s41927-021-00196-1.

## Background

Since the use of biologic agents in the therapeutic armamentarium against rheumatoid arthritis (RA), low disease activity (LDA) and remission are common goals for better outcomes, including less radiographic progression [1]. In clinical trials, remission is often defined as a 28-joint disease activity score (DAS_28_) < 2.6. However, this target is achieved in only 30–40% of patients [2, 3]. Within this group, the disease remains active in a significant proportion of patients, as observed in the DREAM registry, where 31.1% of patients had a swollen joint count ≥2 [3] and experienced joint damage progression [4]. Furthermore, imaging studies have shown at least one synovitis in 33–73% of patients in remission by ultrasound (US) and in up to 96% of them by magnetic resonance imaging (MRI) [5]. Surprisingly, even the most stringent remission criteria, such as ACR/EULAR Boolean remission [6], did not decrease the prevalence of US-diagnosed synovitis [7–11]. Although the relevance of US, specifically power Doppler activity, in clinical remission is widely accepted for driving radiologic progression [8–14] and future clinical flares [7, 10, 13, 15], the relevance of other imaging techniques for assessing synovitis remains poorly understood, and discordance between predictors of clinical and US remission indicates complex interactions between them [9, 16].

We and others [17–24] have shown that 2-[^18^F]fluoro-2-deoxy-D-glucose positron emission computed tomography ([^18^F] FDG PET/CT) is able to detect and quantify inflammation in RA synovitis. The number of PET-positive joints among the 28 joints of the DAS and the cumulative standard uptake value (CSUV) of these PET-positive joints were highly correlated with clinical status: number of swollen and tender joints, erythrocyte sedimentation rate (ESR) and C-reactive protein (CRP) parameters [22, 24] and also with US data such as the number of US-positive joints and the cumulative synovial thickness [22]. Roivanen et al. [21] reported that up to 90% of the joints were rated positively by clinical evaluation (swollen and tender) and also by [^18^F] FDG PET, whereas a proportion of 75% was quoted by Elzinga [19]. However, in these studies, correlations between PET parameters and DAS_28_ scores were obtained in RA patients who had for all [20, 22] or a large majority [21, 24] severe disease activity. The main objectives of the present study were (1) to correlate PET/CT parameters to US and clinical measurements among RA patients and (2) to analyse variations in PET/CT parameters based on disease activity (remission, low/moderate activity or severe activity) defined by two classic composite indices, DAS_28_-CRP and Clinical Disease Activity Index (CDAI) scores.

## Methods

### Study design and patients

This cross-sectional study, approved by the ethics committee of our hospital (B70720108722), included 63 patients fulfilling the ACR/EULAR 2010 criteria for RA [25] from July 2010 to April 2012. Written informed consent was obtained from each patient. All assessments were performed on the same day by the same independent experienced investigator unaware of the other results: the clinical evaluation and biological test were performed in the morning followed by US and then PET/CT evaluation. X-rays were available as routine controls and were performed at a maximum of 6 weeks after the study day. The patient (PtGA) and the physician (PGA) global assessments were determined using a visual analogue scale (VAS) (0–100 mm) as well as the Health Assessment Questionnaire (HAQ) [26]. Disease activity was evaluated using the DAS_28_-CRP (without the PGA) [27] and the CDAI (with the PGA and without CRP) [28]. Each patient was categorized as in remission (DAS_28_-CRP ≤ 2.6 or CDAI≤2.8), in low to moderate disease activity (2.6 < DAS_28_-CRP ≤ 5.1, 2.8 < CDAI≤22), or in severe disease activity (DAS_28_-CRP > 5.1, CDAI> 22) [29]. The number of joints that were solely tender (T), solely swollen (S), “tender or swollen” (T/S) and “tender and swollen” (T&S) was recorded.

### [^18^F] FDG PET/CT imaging

**Fig. 1:**
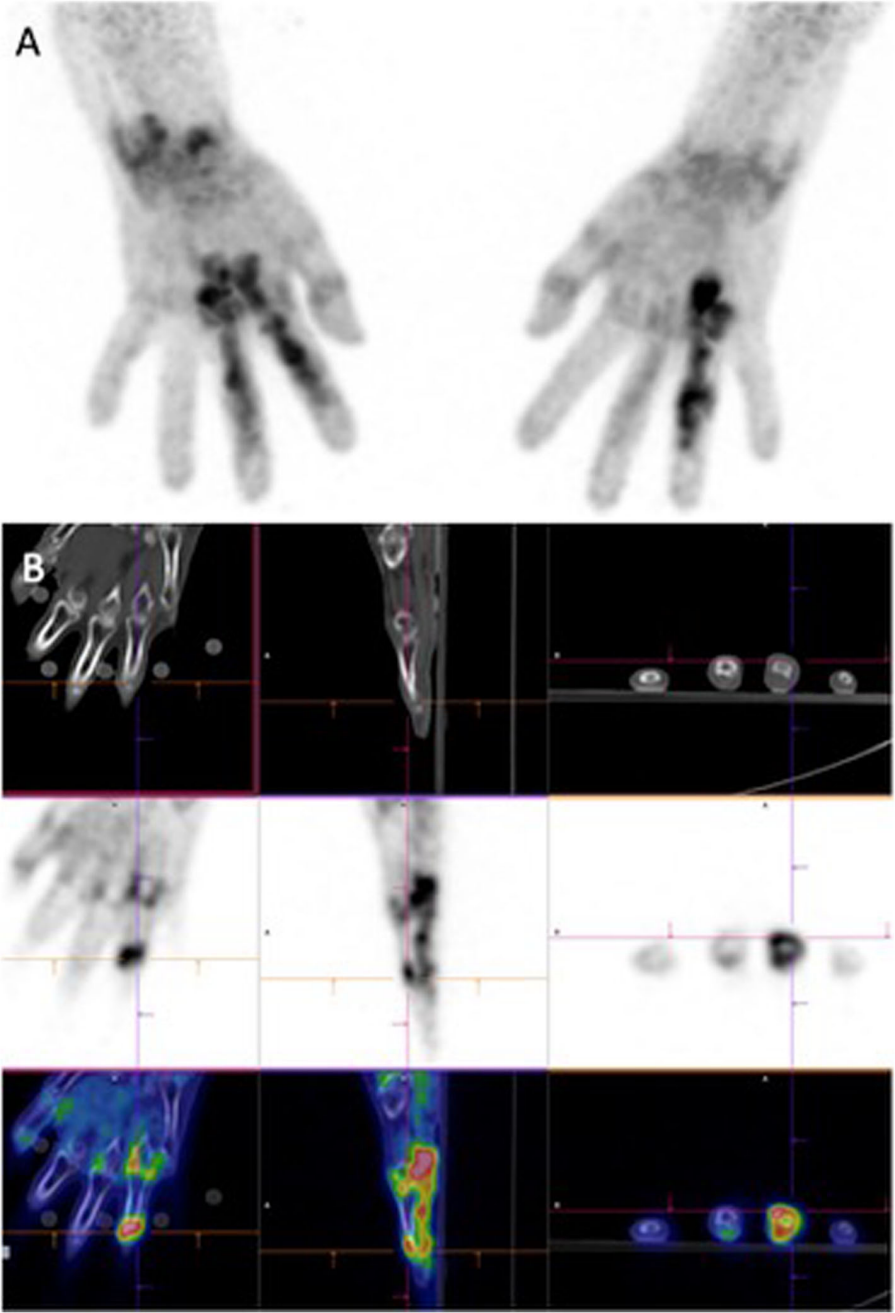
Extensive inflammatory changes in several joints of the hand, along with tenosynovitis. **a**: MIP (maximum intensity projection) of the [^18^F] FDG PET image. **b**: CT (upper row), PET (middle row) and fused PET/CT images (lower row) in the coronal, sagittal and transaxial views, respectively. [^18^F] FDG PET/CT: ^18^F-fluorodeoxyglucose positron emission computed tomography

The PET/CT studies were performed using a Gemini BigBore scanner (Philips Medical Systems, Cleveland, OH, USA). The patients fasted for 4 h and were injected with [^18^F] FDG (4 MBq/kg body weight with a maximum of 370 MBq) through an indwelling catheter placed in the median cubital vein and flushed with 5 cc of saline solution afterwards. Blood glucose levels were lower than 140 mg/dl. The uptake time was 60 min, and the image acquisition sequence was as follows: first, a scout view CT, followed by a PET emission study that included the knees, hands, wrists, elbows and shoulders, with 2 min per bed position for a total scanning time that ranged from 14 to 18 min. Finally, a low-dose CT (5-mm slice thickness, tube voltage 120 Kv, tube current–time product 80 mAs) was performed over these joints. Figure 1 presents an exemplative picture of [^18^F] FDG PET/CT imaging of the knees and hands. The hands and wrists were positioned and fixated, arms down, on a dedicated Plexiglas device to avoid movements between the PET and CT acquisitions. PET images were reconstructed using an iterative list mode time-of-flight algorithm, and corrections for attenuation, dead-time, random and scatter events were applied. The images were first visually analysed, and joints were considered positive for synovitis when the [^18^F] FDG uptake was increased compared to the background in areas corresponding to joint synovium on CT, i.e., either when thickened synovium was recognized on CT or in locations corresponding anatomically to synovium, excluding uptake in other structures such as muscle and tendons. The [^18^F] FDG uptake was then quantified using the maximum standardized uptake value (SUVmax). In PET-positive joints according to the visual analysis, the SUVmax was obtained by drawing a region of interest (ROI) over the most active synovial area identified. When no synovitis was identified, ROIs were placed in the corresponding areas on the CT and drawn around the appropriate joint: at the dorsal surface of the radius (on top of the lunate) for the wrists, over the lateral recess at the level of the midpatella for the knees and for the small joints as metacarpophalangeal (MCP) or proximal interphalangeal (PIP) joints. A global metabolic assessment was obtained through the number of PET-positive joints (visual evaluation) and the sum of all SUVmax values from the positive joints (cumulative SUV, CSUV).

### Ultrasound and X-ray examinations

US assessments were performed using a B-mode multifrequency 10–14.0 MHz transducer (Logiq 9) (GE Healthcare, Milwaukee, WI, USA). US positioning for the wrists, MCP and PIP joints and for the knees has been described elsewhere [22]. Proximal and distal radiohumeral recesses and posterior recess were studied for the elbows. The glenohumeral joint, with a posterior transverse view, was studied for the shoulders. Synovial measurements were systematically carried out perpendicular to the great axis and at the point of greatest thickness. A cut-off for US positivity was defined as synovitis that was at least 1-mm thick (3 mm for the shoulders) based on US determinations in healthy controls, described elsewhere [22]. In joints where 2 (wrists) or 3 (elbows, knees) scans were obtained, the joint was considered positive if at least one measurement was positive. The cumulative synovial thickness (CST), i.e.*,* the sum of thicknesses of all US-positive joints, is the addition of all (single or multiple) synovial measurements performed. X-rays were obtained for peripheral joints (PIPs, MCPs and wrists).

### Statistical analysis

The results are generally expressed as the mean ± standard deviation (SD). Correlation coefficients were calculated to measure the association between PET/CT and clinical or US parameters. Spearman correlations were used for skewed distributions. Concordance between methods was quantified by the intraclass coefficient (ICC). Ordinal logistic regression was used to assess the relationship between disease activity categories based on the DAS_28_-CRP or CDAI (remission, low/moderate and severe disease activity) and PET/CT number of positive joints and CSUV. A test was performed to determine whether all three disease severity categories were distinguishable. If this was not the case, a classic logistic regression analysis was applied, and optimal Youden cut-off values were determined from the receiver operating characteristic (ROC) curve method. The results were considered significant at the 5% level (*p* < 0.05). All statistical analyses were performed with SAS (version 9.4).

## Results

### Patient characteristics

The study patients (42 women and 21 men) had a mean age of 54.8 ± 12.3 years and disease duration of 7.0 ± 6.0 years. IgM rheumatoid factor and anti-citrullinated antibodies were positive in 49.2 and 69.8%, respectively. At baseline, 40 (63.5%) subjects were taking classic disease-modifying antirheumatic drugs (DMARDs); 32 (50.8%), biological agents; 19 (30.2), daily oral prednisolone; and 13 (20.6%), non-steroidal anti-inflammatory drugs. Laboratory, physical examination, disease activity, US and PET/CT results are displayed in Table 1.

**Table 1:** Patient-related and joint-related characteristics of study material. *VAS* Visual Analogue Scale, *CRP* C-reactive protein, *ESR* erythrocyte sedimentation date, *DAS* Disease activity score, *CDAI* clinical disease activity index, *PET/CT* positron emission computed tomography, *SD* standard deviation

Variable	Patient (*N* = 63) Mean ± SD or Number (%)	Joints (*N* = 1764) Number (%)
Anamnestic disease activity		
Patient global assessment - VAS (mm)	43.0 ± 30.6	
Physician global assessment - VAS (mm)	20.1 ± 26.2	
Health Quality Questionnaire	13.3 ± 13.0	
Blood analysis		
CRP (mg/L)	3.3 ± 4.4	
ESR (mm/h)	11.3 ± 12.1	
Clinical examination		
Number of tender joint	5.9 ± 8.2	373 (21.1)
Number of swollen joint	3.8 ± 4.6	242 (13.7)
Number of tender or swollen joint	7.7 ± 8.0	486 (27.6)
Number of tender and swollen joint	2.1 ± 4.3	129 (7.3)
DAS28-CRP	3.4 ± 1.5	
Remission (< 2.6)	22 (34.9)	
Low /moderate activity (2.6–5.1)	31 (49.2)	
Severe activity (> 5.1)	10 (15.9)	
CDAI	16.1 ± 15.2	
Remission (≤2.8)	11 (17.5)	
Low /moderate activity (2.9–22)	37 (58.7)	
Severe activity (> 22)	15 (23.8)	
Ultrasounds		
Synovitis (positive joints)	2.4 ± 3.2	152 (8.6)
Cumulative synovial thickness (mm)	8.3 ± 13.1	
PET/CT		
Positive joints	5.7 ± 7.9	361 (20.5)
Cumulative standard uptake value	12.8 ± 19.1	

### Relationships between PET/CT and US measurements

**Fig. 2:**
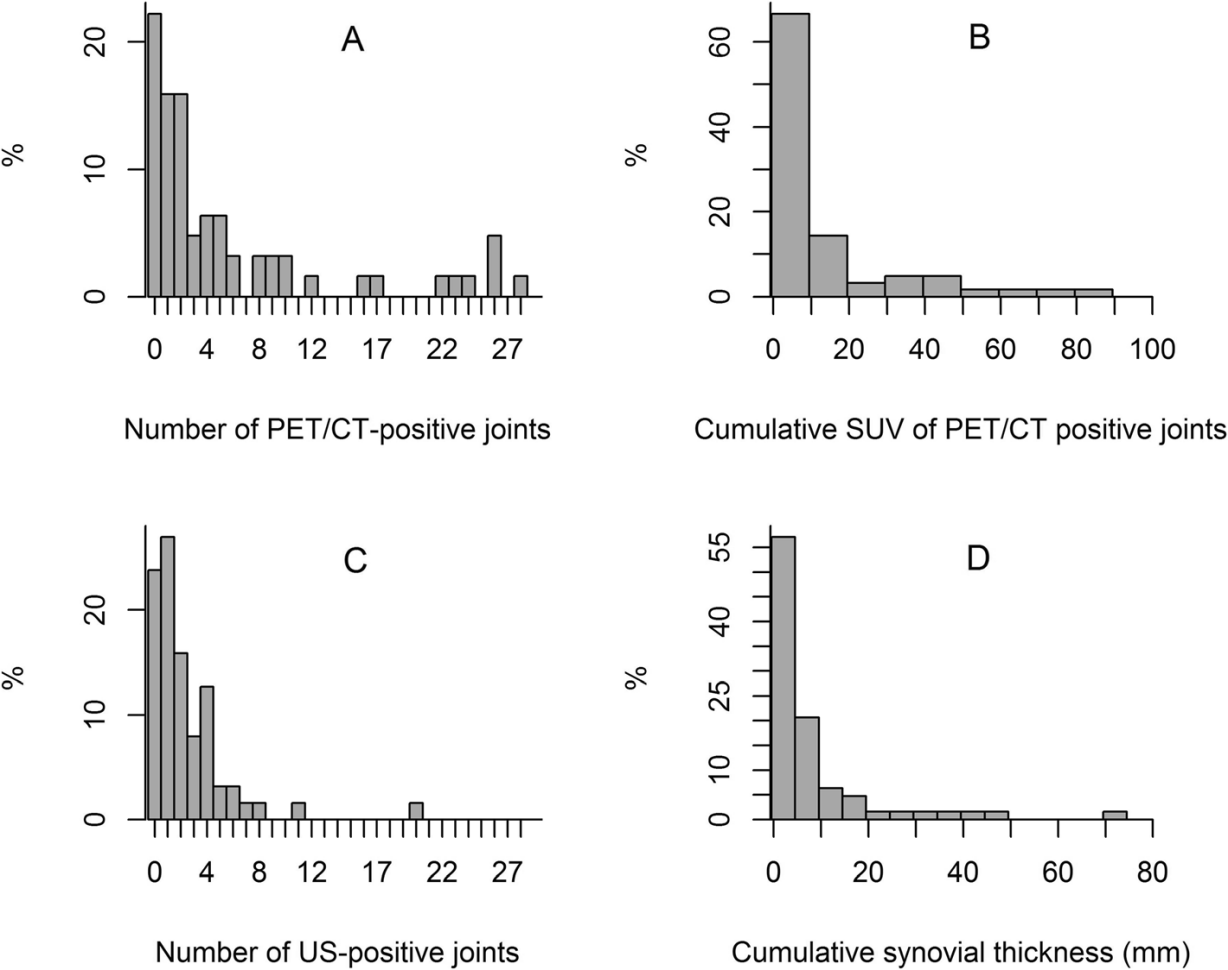
PET/CT and US positivity: distribution of the number of PET-CT-positive joints (**a**), cumulative SUV (**b**), number of US-positive joints (**c**), and cumulative synovial thickness (**d**) in 63 RA patients. PET-CT: positron emission computed tomography; US: ultrasound; SUV: standard uptake value

In total, 1764 (63 × 28) joints were analysed. The number of positive joints and the cumulative activity (CSUV) were obtained by PET/CT, and the number of positive joints and the total synovial thickness (CST) were obtained by US. The distributions of PET/CT- and US-positive joints, CSUV and CST are illustrated in Fig. 2. Significant correlations were found between the number of PET/CT-positive joints and CSUV (*r* = 0.96, *P* < 0.0001) and between the number of US-positive joints and CST (*r* = 0.94, *P* < 0.0001). Significant correlations were also found between the number of PET/CT-positive joints and US measurements (number of PET/CT-positive joints and number of US-positive joints: *r* = 0.42, *P* = 0.0005; number of PET/CT-positive joints and CST: *r* = 0.39, *P* = 0.0017) and between CSUV and US measurements (CSUV and number of US-positive joints: *r* = 0.41, *P* = 0.0009; CSUV and CST: *r* = 0.39, *P* = 0.0017). Concordance between the number of PET/CT-positive joints and the number of US-positive joints, however, was poor (ICC = 0.34; 95% ICC 0.13); PET/CT-positive joints were twice as frequent as US-positive joints.

### Relationships between PET/CT and clinical measurements

Joint positivity on PET/CT was compared to the clinical evaluation (“tender”, “swollen”, “tender or swollen”, “tender and swollen”) for each joint. The diagnostic efficacy of PET/CT (sensitivity, specificity, and positive and negative predictive values) is presented in Table 2. PET/CT sensitivity was low with respect to clinical measurements: only 58.9% of the joints that were both tender and swollen and only 35.8% of the joints that were tender or swollen were positive on PET/CT. Specificity was higher: 82.6% of the joints that were not tender or not swollen, and 85.4% of the joints that were neither tender nor swollen were PET-negative (Table 2). In accordance with these data, the positive predictive value of PET/CT was low, while the negative predictive value was as high as 96.2% in tender and swollen joints. In other words, when PET/CT was negative, the probability that the articulation was not tender and/or not swollen was high; however, when PET/CT was positive, the probability that this articulation was tender and/or swollen was of poor value.

**Table 2:** Diagnostic efficacy of PET/CT in the 1764 joints analyzed. Values are expressed in percent with 95% confidence interval. The item “swollen” includes “swollen only” and “tender and swollen”. The item “tender” includes “tender only” and “tender and swollen”. The item “tender or swollen” includes “tender only”, “swollen only” and “tender and swollen”. The item “tender and swollen” includes only a joint that is simultaneously tender and swollen”

	Tender	Swollen	Tender or Swollen	Tender and swollen
Sensitivity	40.5 (35.5–45.5)	40.9 (34.7–47.1)	35.8 (31.5–40.1)	58.9 (50.4–67.4)
Specificity	84.9 (83.0–86.8)	82.8 (80.9–84.7)	85.4 (83.4–87.3)	82.6 (80.7–84.4)
Positive predictive value	41.8 (36.7–46.9)	27.4 (22.8–32.0)	48.2 (43.0–53.4)	21.1 (16.8–25.3)
Negative predictive value	84.2 (82.3–86.1)	89.8 (88.2–91.4)	77.8 (75.6–79.9)	96.2 (95.2–97.2)

### Relationships between PET/CT parameters and disease activity threshold

**Fig. 3:**
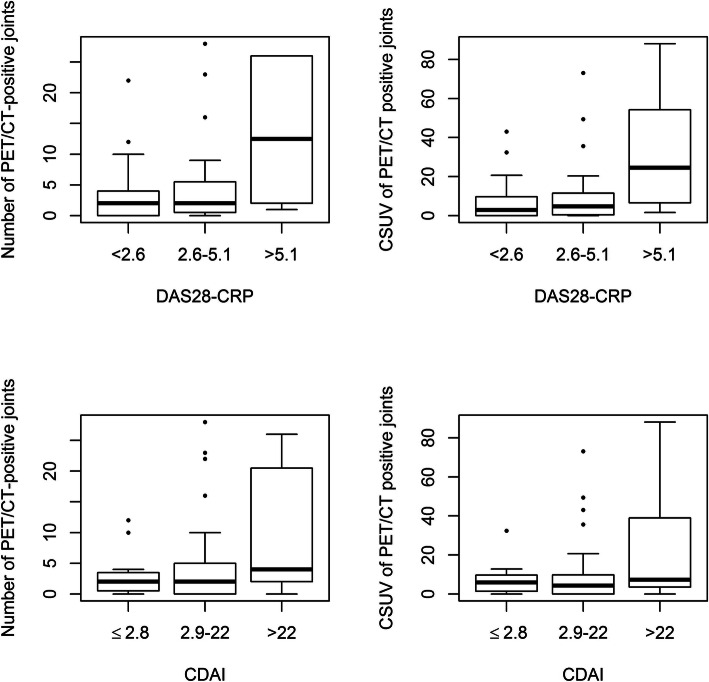
Number of PET/CT-positive joints (left panels) and CSUV (right panels) across disease activity categories based on the DAS_28_-CRP (upper panels) or CDAI (lower panels). PET-CT: positron emission computed tomography; CSUV: cumulative standard uptake value; DAS: disease activity score; CDAI: Clinical Disease Activity Index; CRP: C-reactive protein

The number of PET/CT-positive joints and CSUV were analysed based on disease activity categories (based on the DAS_28_-CRP or CDAI) and illustrated in Fig. 3 and Supplementary Table 1 (e.g., there were 3.6 ± 5.4 PET/CT-positive joints in those with DAS28-CRP remission, 4.7 ± 6.7 joints in those with low/moderate activity, and 13.6 ± 11.2 joints in those with severe disease activity). An ordinal logistic regression evidenced a significant relationship between the mean number of PET/CT-positive joints or the CSUV and clinical disease activity (Supplementary Table 1). However, “remission” and “low/moderate” disease activity categories could not be dissociated by PET/CT. Moreover, 27.3% of patients without any metabolic activity were observed in both remission subgroups defined by the DAS_28_-CRP and CDAI, while 25.8 and 27% of patients were observed in the DAS_28_-CRP and CDAI low/moderate activity subgroups (*p* = 0.99 for both DAS_28_-CRP and CDAI subgroups), indicating that PET/CT was unable to discern remission and low/moderate activity (Table 3).

**Table 3:** Number of patients with PET/CT-negative joints in remission and low/moderate activity. *DAS* disease activity score, *CRP* C-reactive protein, *CDAI* clinical disease activity index, *PET/CT* positron emission computer tomography

	DAS_28_-CRP		CDAI	
	N	Number of patients with PET/CT = 0 (%)	N	Number of patients with PET/CT = 0 (%)
Remission	22	6 (27.3)	11	3 (27.3)
Low/moderate	31	8 (25.8)	37	10 (27.0)
*P*-value (chi-square)		0.99		0.99

Thus, the two categories remission and low/moderate activity were merged, and a classic logistic regression analysis was performed between patients with severe and non-severe (including remission and low/moderate activity) disease activity (Table 4): highly significant differences were found in the number of PET/CT-positive joints and in CSUV. The optimal threshold for identifying RA patients with clinically and biologically severe disease was at least 8 PET/CT-positive joints and a CSUV ≥17.8 for the DAS_28_-CRP and 6.8 and 15.0 for the CDAI, respectively. Disease activity thresholds were also studied by dividing RA patients into remission and non-remission categories (including low/moderate and severe disease), but no significant differences were observed in terms of the number of PET/CT-positive joints and CSUV (data not shown).

**Table 4:** Association between the number of PET/CT-positive joints or cumulative SUV and disease activity based on DAS_28_-CRP or CDAI. *DAS* disease activity score, *CRP* C-reactive protein, *CDAI* clinical disease activity index, *PET/CT* positron emission computer tomography, *SUV* standard uptake value, *AUC* area under the curve, *SD* standard deviation

Clinical disease activity	N	Number of PET/CT- positive joints			Cumulative SUV		
		Mean ± SD	Cut-off (AUC)	*P*-value	Mean ± SD	Cut-off (AUC)	*P*-value
DAS_28_-CRP			8.0 (0.77)	0.0026		17.8 (0.77)	0.0046
Non-severe≤5.1	53	4.2 ± 6.1			9.2 ± 14.1		
Severe> 5.1	10	13.6 ± 11.2			31.9 ± 29.9		
CDAI			6.8 (0.67)	0.023		15.0 (0.65)	0.033
Non-severe≤22	48	4.4 ± 6.3			9.7 ± 14.6		
Severe> 22	15	10.0 ± 10.6			22.9 ± 27.5		

Table 3 shows that 16/22 and 8/11 patients in remission (according to DAS_28_-CRP and CDAI, respectively) were nonetheless PET/CT-positive for at least one joint. Of interest, 12/22 (54.5%) and 6/11 (54.5%) patients also had at least one US-positive joint, despite being in remission (data not shown). As an illustration regarding the patients in remission based on the DAS_28_-CRP, at the joint level, 75 joints were PET/CT-positive, and 24 were US-positive. Moreover, in the 6 patients with strict Boolean remission (tender and joint score ≤ 1, PGA ≤ 1 (0–10 cm), CRP ≤ 1 mg/dl), 20/168 joints were PET/CT-positive, and 9/168 were US-positive, suggesting a dissociation of PET/CT metrology and the clinical assessment in RA patients in remission. Among the patients in remission based on the DAS_28_-CRP, only 10 joints were positive for both PET/CT and US (5 wrists, 4 MCPs, and 1 shoulder). The 65 remaining PET/CT-positive joints were distributed as follows: 27 PIPs, 21 MCPs, 8 wrists, 7 knees, 1 elbow and 1 shoulder. Among the 6 patients under Boolean remission, only 4 wrists were both PET/CT- and US-positive, enhancing the lack of concordance between PET/CT and US measures.

Overall, there was a significant correlation between the metabolic measurements (number of positive joints and CSUV) and the clinical assessments (DAS_28_-CRP and CDAI) (Table 5). However, when classifying patients in remission, low/moderate or severe categories, this significant correlation between PET and clinical assessment was observed only in the subjects with severe activity according to CDAI and not in the RA patients in the low/moderate disease activity or remission groups.

**Table 5:** Correlation coefficient (with *P*-value) between the number of PET/CT-positive joints or the cumulative SUV and clinical scores (*DAS* disease activity score, *CRP* C-reactive protein, *CDAI* clinical disease activity index, *PET/CT* positron emission computer tomography. *CSUV* cumulative standard uptake value)

	Global	Remission	Low/moderate	Severe
PET/CT-positive joints				
DAS_28_-CRP	0.34 (0.0024)	0.22 (0.33)	−0.12 (0.53)	0.17 (0.63)
CDAI	0.44 (0.0004)	−0.21 (0.54)	0.09 (0.61)	0.58 (0.024)
CSUV				
DAS_28_-CRP	0.37 (0.0028)	0.24 (0.28)	−0.14 (0.44)	0.22 (0.55)
CDAI	0.44 (0.0003)	−0.07 (0.85)	0.06 (0.71)	0.63 (0.012)

### Relationship between X-rays and PET/CT

The 63 peripheral joints (PIPs, MCPs and wrist) that were PET-positive but US-negative in the 16 patients in clinical remission (DAS28-CRP < 2.6) were characterized with X-ray. Features of RA, i.e., symmetrical joint narrowing, bone erosion or demineralization, and of OA, i.e., asymmetrical joint narrowing, subchondral sclerosis, or osteophytes, were recorded. The results were consistent with RA in 24/63 joints (38,1%) and with OA in 8/63 joints (12.7%) with OA signs. In 31/63 joints (49.2%), X-rays were normal. In particular, RA/OA/normal features were described in 7/0/24 of the 31 PIPs, 12/6/5 of the 23 MCPs and 5/2/2 of the 9 wrists. Considering the corresponding clinical status, none of these joints were tender or swollen.

## Discussion

In line with previous work [22], we confirmed that the number of PET/CT-positive joints and the CSUV significantly correlated with the number of US-positive joints, synovial thickness and disease activity based on either the DAS_28_-CRP or CDAI. In addition, PET/CT was quite effective at distinguishing patients with a severely active disease from the others, as a cut-off of 8 for the number of PET-positive joints and 17.8 for the CSUV yielded an area under the curve (AUC) of 0.77 (considering the DAS_28_-CRP as the clinical gold standard). Although the number of hypermetabolic joints and the cumulative SUV tended to be higher with increased clinical severity of the disease, one notable exception should be mentioned. There was no significant difference with the number of PET-positive joints and their CSUV between patients in clinical remission and those with low/moderate disease activity. In both groups, only 25–27% of the patients presented negative PET/CT findings. Clearly, PET/CT results and clinical assessments diverge in non-severe RA, including remission, in agreement with previous observations made with US and MRI [3–9].

Comparing the PET and US findings, there were twice as many PET/CT-positive joints as US-positive joints. Furthermore, there was also clear evidence that the PET/CT and US analyses of joints did not concur for those in remission. For example, out of the 22 patients in remission based on the DAS_28_-CRP, 12 were positive with both PET/CT and US, but at the joint level, only 10 of the 75 PET/CT-positive joints (5 wrists, 4 MCPs and one shoulder) were also US-positive. This divergence was also observed for patients in remission according to the strict Boolean-based definition (which is stricter than the DAS_28_-CRP-based definition). In other words, PET/CT was positive in a significant number of patients with no or low/moderate disease activity based on current clinical scales and was also positive in a significant number of joints that were not considered inflamed according to clinical and US parameters. Two interpretations are possible for this observation. The first would be a higher sensitivity of the metabolic measurements for identifying subclinical joint inflammation. Indeed, in inflammatory diseases, incidental PET/CT findings due to [^18^F] FDG accumulation are consistently associated with enhanced glycolytic metabolism in inflammatory cellular infiltrates, including activated macrophages, neutrophils and lymphocytes [30]. We may therefore consider that hypermetabolic joints with normal US appearance are joints with an inflammatory component without proliferating synovitis or with a synovitis < 1 mm thickness, which was the cut-off. In a previous series of RA patients with severe disease activity, only 50% of the PIPs and 62% of the MCPs, both tender and swollen, were US-positive using the same cut-off (data not shown) [22]. PET/CT analysis might therefore exhibit greater sensitivity than US. It is noteworthy that in the current series, the PET/CT-positive but US-negative joints within these 16 patients were mostly PIPs (31 joints in 7 patients) and MCPs (23 joints in 7 patients), which are joints that are typically involved in RA. X-ray analysis supports this hypothesis, as 38% of the joints had signs of RA and 49% were normal. An alternative explanation would be to consider those joints and patients as false positive results of PET/CT. It is indeed possible that the joints actually suffer from secondary (MCPs) or primary (PIPs) osteoarthritis. However, only 8/62 (13%) joints, 6 MCPs in 2 patients and 2 wrists in 2 patients had signs of OA. The radiological analysis is thus in favour of the first hypothesis, but a longitudinal follow-up of the patients would be needed to provide definitive evidence. As a limitation, X-rays were only available for peripheral joints (PIPs, MCPs and wrist) with systematic X-ray realization but not for larger joints (knee, elbow, shoulder). In addition, it should be noted that 30% of the studied patients had daily oral glucocorticoid intake, even at low doses, and that our population was composed of 35% of patients in remission (based on the DAS28-CRP) and only 16% of patients with severe activity; these factors could have influenced our results. A brief report also proposed the use of hybrid PET/magnetic resonance imaging (PET-MRI) to assess inflammatory changes within the synovial tissue in RA [31]. This technique could also be promising to assess remission, mainly because of a higher spatial resolution but also because of the capacity of MRI to detect bone marrow oedema, which is a sign of activity and a risk factor for progression. However, RA was not restricted to the hand, and the use of PET/CT allowed us to obtain a CT view of several other joints, such as the knees, elbows and shoulders.

## Conclusion

[^18^F] FDG PET/CT demonstrated a high specificity and negative predictive value compared to individual clinical evaluation of the joints. Furthermore, PET/CT was effective at differentiating “severe” from “non-severe” patients, although clinical remission was not associated with metabolic remission. Such issues are of high clinical relevance, as PET/CT could possibly identify subclinical and infra-radiological inflammation worthy of treatment to prevent further irrevocable damage to the joints. Further studies are needed to ascertain whether this represents a clinically relevant activity of the disease or secondary degenerative changes.

## Supplementary Information

**Additional file 1: Supplementary Table 1.** Association between the number of PET/CT-positive joints or cumulative uptake value and disease activity based either on DAS_28_-CRP or CDAI by ordinal logistic regression. PET/CT: positron emission computer tomography. CSUV: cumulative standard uptake value. DAS: disease activity score. CRP: C-reactive protein. CDAI: clinical disease activity index.

## Data Availability

The datasets used and/or analyzed during the current study are available from the corresponding author on reasonable request.

## Abbreviations

RA: rheumatoid arthritis
LDA: low disease activity
DAS_28_: Disease activity score 28-joint
US: Ultrasonography
MRI: Magnetic resonance imaging
^18^F-FDG PET/CT: ^18^F-Fluorodeoxyglucose Positron Emission Computed Tomography
CSUV: Cumulative standard uptake values
ESR: Erythrocyte sedimentation rate
CRP: C-reactive protein
CDAI: Clinical Disease Activity Index
PtGA: Patient Global Assessment
PGA: Physician Global Assessment
VAS: Visual Analogue Scale
HAQ: Health Assessment Questionnaire
T: Tender
S: Swollen
T/S: Tender or swollen
T&S: Tender and swollen
SUVmax: Maximum standardized uptake value
ROI: Region of interest
MCP: Metacarpophalangeal
PIP: Proximal interphalangeal
CST: Cumulative synovial thickness
SD: Standard deviation
ICC: Intraclass correlation coefficient
ROC: Receiver Operating Characteristic
DMARDs: Disease-modifying antirheumatic drugs
ICC95%: 95% lower limit confidence
AUC: Area under the curve
