# Supplementary material for: Dissociation between 2-[18F]fluoro-2-deoxy-D-glucose positron emission computed tomography, ultrasound and clinical assessments in patients with non-severe rheumatoid arthritis, including remission
Source: BMC Rheumatol. 2021 Aug 4;5:31. doi: 10.1186/s41927-021-00196-1 (PMC8336401; doi:10.1186/s41927-021-00196-1)
Supplement: Supplementary file 1 — Additional file 1: Supplementary Table 1. Association between the number of PET/CT-positive joints or cumulative uptake value and disease activity based either on DAS28-CRP or CDAI by ordinal logistic regression. PET/CT: positron emission computer tomography. CSUV: cumulative standard uptake value. DAS: disease activity score. CRP: C-reactive protein. CDAI: clinical disease activity index. [file 41927_2021_196_MOESM1_ESM.docx]

SUPPLEMENTARY MATERIAL

**Supplementary Table 1:** Association between the number of PET/CT-positive joints or cumulative uptake value and disease activity based either on DAS_28_-CRP or CDAI by ordinal logistic regression. PET/CT: positron emission computer tomography. CSUV: cumulative standard uptake value. DAS: disease activity score. CRP: C-reactive protein. CDAI: clinical disease activity index.

| Clinical disease activity | N | Number of PET/CT- positive joints | | CSUV | |
| --- | --- | --- | --- | --- | --- |
|  |  | Mean ± SD | P-value | Mean ± SD | P-value |
| DAS28-CRP | |  | 0.0034 |  | 0.0055 |
| Remission <2.6 | 22 | 3.6 ± 5.4 |  | 7.9 ± 11.1 |  |
| Low/moderate 2.6-5.1 | 31 | 4.7 ± 6.7 |  | 10.2 ± 16.0 |  |
| Severe>5.1 | 10 | 13.6 ± 11.2 |  | 31.9 ± 29.9 |  |
| CDAI | |  | 0.022 |  | 0.033 |
| Remission ≤2.8 | 11 | 3.3 ± 4.0 |  | 7.6 ± 9.4 |  |
| Low/moderate 2.9-22 | 37 | 4.7 ± 6.9 |  | 10.3 ± 15.9 |  |
| Severe>22 | 15 | 10.0 ± 10.6 |  | 22.9 ± 27.5 |  |
